# Highly variable timing renders immunotherapy efficacy and toxicity impractical biomarkers of one another in clinical practice

**DOI:** 10.3389/fimmu.2024.1351739

**Published:** 2024-04-16

**Authors:** Mitchell S. von Itzstein, Yuqiu Yang, Yiqing Wang, David Hsiehchen, Thomas Y. Sheffield, Farjana Fattah, Vinita Popat, Murtaza Ahmed, Jade Homsi, Jonathan E. Dowell, Sawsan Rashdan, Jay Lohrey, Hans J. Hammers, Randall S. Hughes, Tao Wang, Yang Xie, David E. Gerber

**Affiliations:** ^1^Department of Internal Medicine (Division of Hematology-Oncology), Dallas, TX, United States; ^2^Harold C. Simmons Comprehensive Cancer Center, University of Texas Southwestern Medical Center, Dallas, TX, United States; ^3^Department of Population and Data Sciences, University of Texas Southwestern Medical Center, Dallas, TX, United States; ^4^School of Medicine, University of Texas Southwestern Medical Center, Dallas, TX, United States

**Keywords:** efficacy, immune checkpoint inhibitor (ICI), immune-related adverse event (irAE), immunotherapy, monitoring, toxicity, biomarker

## Abstract

**Background:**

A useful clinical biomarker requires not only association but also a consistent temporal relationship. For instance, chemotherapy-induced neutropenia and epidermal growth-factor inhibitor-related acneiform rash both occur within weeks of treatment initiation, thereby providing information prior to efficacy assessment. Although immune checkpoint inhibitor (ICI)-associated immune-related adverse events (irAE) have been associated with therapeutic benefit, irAE may have delayed and highly variable onset. To determine whether ICI efficacy and irAE could serve as clinically useful biomarkers for predicting each other, we determined the temporal relationship between initial efficacy assessment and irAE onset in a diverse population treated with ICI.

**Methods:**

Using two-sided Fisher exact and Cochran-Armitage tests, we determined the relative timing of initial efficacy assessment and irAE occurrence in a cohort of 155 ICI-treated patients (median age 68 years, 40% women).

**Results:**

Initial efficacy assessment was performed a median of 50 days [interquartile range (IQR) 39-59 days] after ICI initiation; median time to any irAE was 77 days (IQR 28-145 days) after ICI initiation. Median time to first irAE was 42 days (IQR 20-88 days). Overall, 58% of any irAE and 47% of first irAE occurred after initial efficacy assessment. For clinically significant (grade ≥2) irAE, 60% of any and 53% of first occurred after initial efficacy assessment. The likelihood of any future irAE did not differ according to response (45% for complete or partial response vs. 47% for other cases; *P*=1). In landmark analyses controlling for clinical and toxicity follow-up, patients demonstrating greater tumor shrinkage at initial efficacy assessment were more likely to develop future grade ≥2 (*P*=0.05) and multi-organ (*P*=0.02) irAE.

**Conclusions:**

In contrast to that seen with chemotherapy and molecularly targeted therapies, the temporal relationship between ICI efficacy and toxicity is complex and bidirectional. In practice, neither parameter can be routinely relied on as a clinical biomarker to predict the other.

## 1 Introduction

For decades, clinicians and researchers have recognized an association between efficacy and toxicity of systemic cancer therapies. With cytotoxic chemotherapy, the development of early cytopenias is correlated with subsequent treatment benefit (1–5), thereby serving as a potential predictive biomarker. Similar efficacy-toxicity relationships have emerged for molecularly targeted therapies, including better outcomes in the setting of high-grade cutaneous reactions from epidermal growth factor receptor (EGFR) inhibitors (6–10) or hypertension from antiangiogenic agents (11, 12).

More recently, a clear relationship between immune-related adverse events (irAE) and improved efficacy has emerged in populations treated with immune checkpoint inhibitors (ICI) (13–19). Distinct from the toxicity-efficacy relationships observed with cytotoxic and targeted cancer therapies, this association has been attributed to bystander effects from activated T cells, essentially serving as a surrogate marker of robust anti-tumor immune responses.

irAE also differ from common toxicities of other systemic cancer treatments in another key aspect: their timing. Generally, chemotherapy and targeted therapy adverse effects occur early during treatment. For most cytotoxic agents, blood count nadir occurs within two weeks after chemotherapy is started (20–23). EGFR inhibitor-induced rash generally appears within 7-10 days of treatment initiation (24, 25). By contrast, irAE may emerge months after treatment initiation, including well after treatment is stopped in some cases (26–28).

The potential for variable and delayed onset of irAE has clear clinical implications. Because chemotherapy- or targeted therapy-related toxicities usually occur before efficacy assessment, these events may serve as clinical biomarkers predicting future therapeutic benefit. However, later toxicities such as irAE might follow rather than precede initial efficacy evaluation. In such cases, irAE would not serve as useful clinical biomarkers of ICI efficacy. Instead, ICI efficacy might predict future irAE, a scenario that could allow clinicians and patients to modify monitoring and expectations. To evaluate the potential utility of ICI efficacy and toxicity as clinical biomarkers of each other, we evaluated the temporal relationship between ICI efficacy and irAE occurrence in a diverse cohort of patients treated with ICI.

## 2 Methods

### 2.1 Patient selection and study procedures

**Figure 1:**
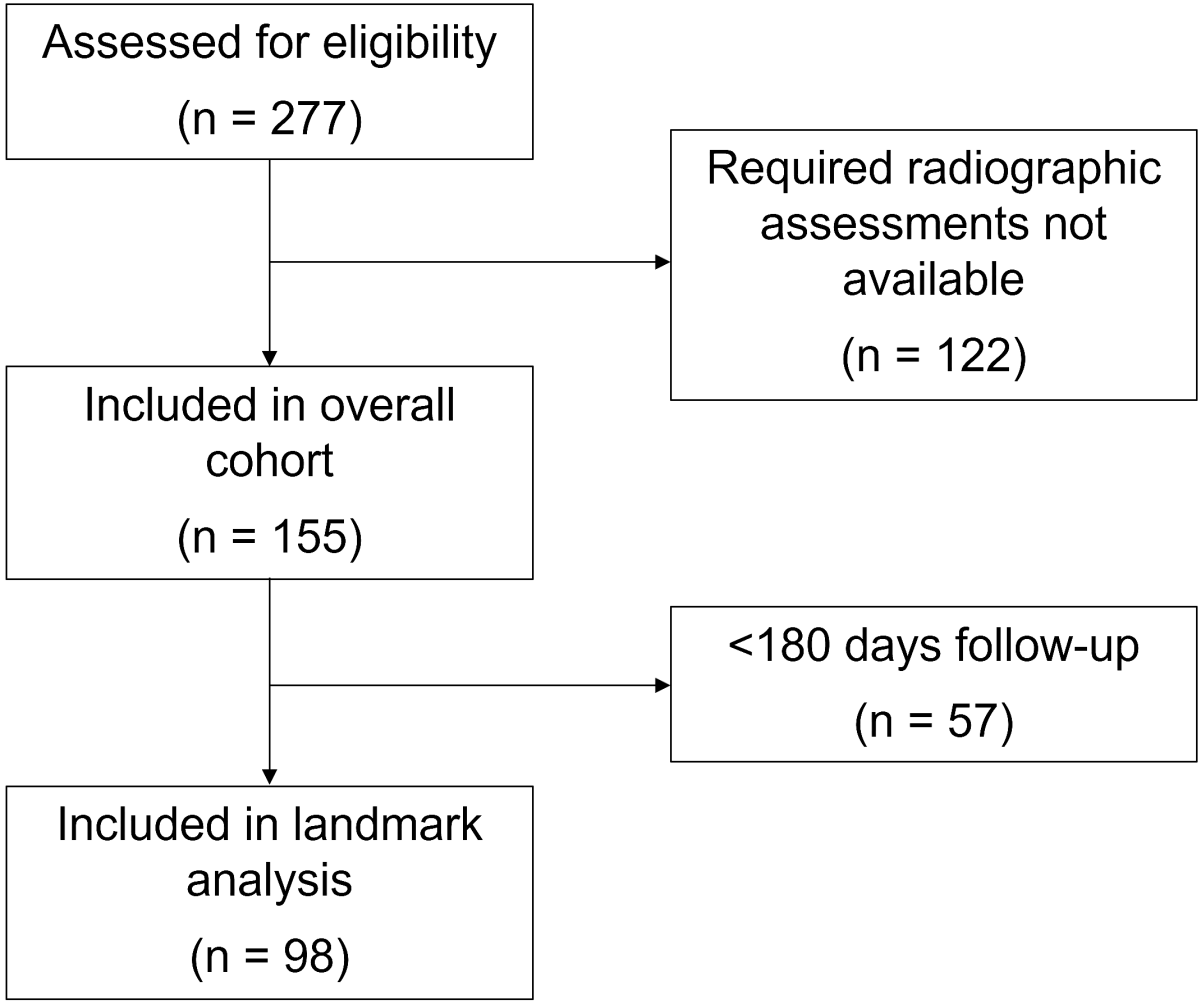
Consort flow diagram.

This study was conducted within a prospective registry of cancer immunotherapy approved by the UT Southwestern Institutional Review Board (IRB #STU 082015-053). All methods were carried out in accordance with institutional guidelines and in accordance with the Declaration of Helsinki. Written and verbal informed consent was obtained from all participants. As previously described (19, 29, 30), we identified patients with a confirmed cancer diagnosis who initiated ICI therapy (PD1, PD-L1 and CTLA4 inhibitors) for active disease between November 2015 (registry initiation) and December 2020 at the Harold C. Simmons Comprehensive Cancer Center at UT Southwestern. To obtain the required data for analysis, identifiable relevant medical records were abstracted between November 2015 and December 2021. Other key inclusion criteria included no prior treatment with ICI therapy and availability of serial radiographic studies to assess response (**Figure 1**). Based on this last requirement, we included only individuals with advanced (stage 4) disease.

### 2.2 Clinical data collection and characterization

We collected the following data from enrolled subjects: demographics (age, sex, race), cancer type, type and dates of ICI therapy, radiographic response, and irAE (onset, type, grade). For efficacy assessments, we determined the percent change between the baseline tumor assessment imaging study and the first imaging study after ICI initiation. The baseline imaging assessment was defined as the latest cross-sectional imaging study performed between 8 weeks before ICI initiation and—if no imaging studies were performed in that interval—up to 3 weeks after ICI initiation. We used the first imaging study performed 3 to 12 weeks after ICI initiation as the first post-ICI initiation efficacy. Additionally, we excluded cases for which the baseline and first post-ICI scan occurred within 3 weeks of each other, as this interval is unlikely to be sufficient to assess efficacy. Because the assessment of irAE occurrence, type, and timing is known to vary among clinicians (31), two separate clinical reviewers (M.S.v.I. and D.H.) assessed each case to determine irAE status. Discrepancies were reviewed and adjudicated by these reviewers and a third clinician with expertise in ICI administration and monitoring (D.E.G.). Review of radiographic response was performed blinded to irAE data, and vice versa.

### 2.3 Statistical analysis

We determined the temporal association between initial efficacy assessment and (a) any irAE and (b) first irAE occurrence using two-sided Fisher exact and Cochran-Armitage tests. Standard alpha of 0.05 was used to determine significance. To control for timing bias (e.g., patients who do not benefit from ICI receive may live for shorter periods and therefore have lower risk of irAE), we performed a landmark analysis including patients who remained alive, had available follow-up for at least 180 days after ICI initiation, and considering irAE occurrence up to 180 days after ICI initiation. All computation was performed using R (v4.1.2).

## 3 Results

A total of 155 patients were included in the study. Median age was 68 years and 62 (40%) were women. Additional demographic and clinical characteristics are shown in **Table 1**. Within the cohort, 140 patients (90%) had baseline imaging studies prior to the date of ICI initiation; these were performed a median 14 days [interquartile range (IQR) 9-27 days] before the first ICI dose. Fifteen patients (10%) had baseline imaging studies performed after ICI initiation [median 11 days (IQR 6-14 days) after first ICI dose (**Supplementary Figure 1**)]. Post-ICI initiation imaging studies, available for all patients in the study, were performed a median of 50 days after ICI initiation (IQR 39-59 days) (**Supplementary Figure 2**). The median interval between baseline and first post-ICI initiation imaging study was 64 days (IQR 49-84 days).

**Table 1:** Characteristics of 155 cases included in the analysis.

Characteristic	Median age (range) or n (%)
Age	68 (27–89)
Sex Female Male	62 (40) 93 (60)
Race Asian Black White Unknown	6 (4) 14 (9) 128 (83) 7 (4)
Cancer type Head and neck Kidney Lung Melanoma Other	7 (4) 7 (4) 111 (72) 13 (8) 17 (11)
ICI type Anti-PD1/PDL1 Anti-PD1/PDL1 + anti-CTLA4 Other*	115 (72) 19 (12) 24 (15)

*Includes CTLA4 monotherapy and other ICI combinations.

Overall, 98 patients (63%) developed any grade irAE (**Table 2**). These events occurred a median of 77 days (IQR 28-145 days) after ICI initiation, with first irAE occurring a median of 42 days (IQR 20-88 days) after ICI initiation. Clinically significant (grade ≥2) irAE occurred in 62 patients (40%) at a median of 83 days (IQR 31-158 days) after ICI initiation, with first grade ≥2 irAE occurring a median of 40 days (IQR 22-82 days) after ICI initiation.

**Table 2:** Occurrence and timing of irAE.

irAE type	Number (%)	Time to onset (d) [median (IQR)]
Colitis/diarrhea	16 (10)	110 (58–260)
Dermatitis/Pruritus/Rash	29 (19)	56 (25–120)
Hepatitis	37 (24)	38 (14–100)
Hyperthyroidism	12 (7)	52 (28–110)
Hypophysitis	5 (3)	110 (69–130)
Hypothyroidism	31 (20)	83 (40–140)
Nephritis	10 (6)	110 (84–200)
Pancreatitis	4 (3)	68 (37–96)
Pneumonitis	35 (23)	82 (41–170)
Other	26 (14)	59 (28–200)

Response Evaluation Criteria in Solid Tumors (RECIST) radiographic response according to initial efficacy assessment was as follows: complete response (CR) (n=2, 1%), partial response (n=26, 17%), stable disease (SD) (n=94, 61%), and progressive disease (PD) (n=33, 21%).

**Figure 2:**
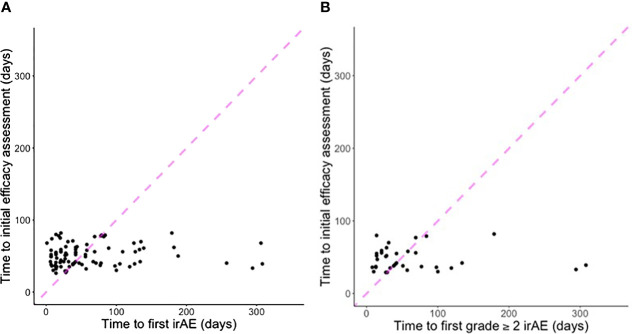
Scatter plots indicating time to first efficacy assessment according to **(A)** first irAE of any grade (five cases with first grade ≥2 irAE after 300 days not shown); **(B)** first grade ≥2 irAE (two cases with first grade ≥2 irAE after 300 days not shown). Points to the left of the dashed line indicate first irAE occurred before first efficacy assessment. Points to the right of the dashed line indicate first efficacy assessment occurred before first irAE.

Overall, 58% of all (and 47% of first) irAE occurred after initial efficacy assessment. For grade ≥2 irAE, 60% of all (and 53% of first) occurred after initial efficacy assessment. **Figure 2** displays the temporal relationship of these events. **Table 3** displays the temporal association between initial efficacy assessment and initial irAE occurrence according to RECIST. The likelihood of any future irAE did not differ by radiographic response (45% for CR/PR vs. 47% for other cases; *P*=1).

**Table 3:** Temporal relationship between first efficacy assessment and first irAE occurrence according to response.

Timing of initial efficacy assessment	Initial efficacy (RECIST)		Total
	Non-CR/PR	CR/PR	
**On or before first irAE**	37	9	46
**After first irAE**	41	11	52
**Total**	78	20	98

Chi-square p=1.0. CR, complete response; ICI, immune checkpoint inhibitor; irAE, immune related adverse event; PR, partial response; RECIST, Response Evaluation Criteria in Solid Tumors.

**Figure 3:**
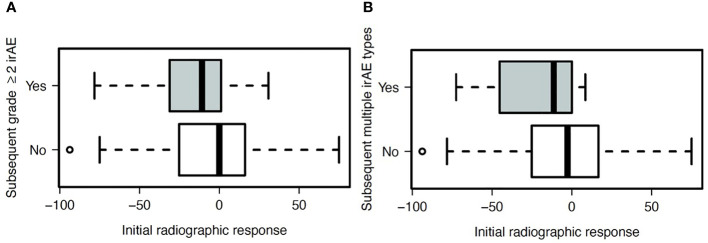
Association between radiographic response on initial ICI efficacy assessment and subsequent development of **(A)** grade ≥2 irAE (*P*=0.05), and **(B)** two or more unique types of irAE (*P*=0.02).

Because initial efficacy assessment may not capture eventual best response from ICI, we also evaluated efficacy as a continuous variable (**Figure 3**). In these analyses, we focused on subsequent occurrence of (a) subsequent occurrence of clinically significant (grade ≥2) irAE and (b) multiple types of irAE, as such cases are more likely to affect patient management. To account for timing bias (e.g., patients who do not benefit from ICI receive may live for shorter periods and therefore have lower risk of irAE), we included only those patients who survived and had available follow-up for 180 days, and we included only those irAE occurring within 180 days. We found that patients with better initial radiographic responses were more likely to develop subsequent grade ≥2 (*P*=0.05) and multiple types (*P*=0.02) of irAE.

## 4 Discussion

The identification of patients most likely to benefit from ICI represents one of the most studied aspects of cancer immunotherapy. To date, the most established predictive laboratory biomarkers reflect tumor biology, including programmed death 1 ligand (PD-L1) expression, microsatellite instability, and mutational burden (32–36). Certain clinical biomarkers are also associated with efficacy of ICI, such as age, sex, body mass index, and exposure to concomitant medications including steroids and antibiotics, and smoking history (36–39). Additionally, it has become clear that individuals who develop immunotherapy-associated toxicities have greater responses, longer disease control, and better survival (13–16).

In recent years irAE have gained recognition as clinically important autoimmune toxicities that are far more varied and less predictable than toxicities of other systemic cancer therapies such as conventional chemotherapy and molecularly targeted therapies. Accordingly, researchers have sought to determine biomarkers for the prediction of irAE, which could influence the selection of patients, treatments, and monitoring. Among others, potential candidates include numerous blood-based parameters such as RNA sequencing, T cell receptor characterization, autoantibodies, cytokines, and immune cell populations (29, 39–41). To date, the clinical characteristic most clearly associated with increased risk of irAE is history of autoimmune disease, which are reported in a substantial proportion of individuals with cancer (42, 43).

Because the onset of irAE can be far more variable and delayed than the toxicities of conventional chemotherapy and molecularly targeted therapy, it is not known whether they could serve as a truly useful clinically biomarker for efficacy. Indeed, it seems conceivable that the converse might be true, with efficacy predicting future irAE. Given these considerations, in the present study we determined the temporal association of initial efficacy assessment and first irAE occurrence—to our knowledge the first study to do so despite the growing number of reports describing an association between these clinical occurrences. In this analysis of more than 150 patients treated with ICI, we observed a complex and bidirectional temporal association between immunotherapy efficacy and toxicity, with about half of first irAE occurring before initial efficacy assessment and half occurring after. In those cases with efficacy apparent before toxicity, patients with better responses were more likely to develop future clinically significant irAE, even when controlling for duration of follow-up. Despite statistical significance, however, the actual difference in irAE risk was quite modest, suggesting that radiographic response is unlikely to serve as a clinically useful biomarker for future irAE risk.

To place our findings in context, it is worth considering the converse scenario. Because chemotherapy and molecularly targeted therapy toxicities almost always emerge early before efficacy is known, adverse events may influence expectations of benefit (1–3, 6–10). In some cases, their absence may even drive treatment modifications geared toward increasing efficacy. For instance, clinical trials have examined the role of escalating EGFR inhibitor dose to achieve moderate acneiform rash (44). Others have evaluated chemotherapy dose escalation to achieve high-grade cytopenias (45, 46).

Currently, expert guidelines for irAE monitoring generally apply a single approach to all patients over the entire course of ICI treatment. For instance, the Society for Immunotherapy of Cancer (SITC) recommends serial history and physical, blood counts, chemistries, and thyroid function tests throughout ICI therapy (47). Our findings highlight the importance of continuing these assessments throughout ICI treatment, as irAE timing remains highly unpredictable. It is not clear that intensified monitoring for individuals who have initial radiographic response would be clinically useful. Alternatively, in the setting of apparent radiographic benefit, stopping or reducing the intensity of ICI treatment to avoid future irAE seems both unethical and counterintuitive, and is not supported by our study results. Given the associated toxicities and hypothetical effects on ICI efficacy, nor does prophylactic administration of corticosteroids seem appropriate.

Our current observations reflect not only the relatively late onset of ICI toxicities, but also the relatively early emergence of clinical and radiographic benefit. Early in the era of contemporary cancer immunotherapy, these treatments appeared to have delayed efficacy. In the registrational trial of sipuleucel-T, an autologous cellular vaccine for metastatic castration-resistant prostate cancer, the overall survival trajectory did not separate from that of placebo until almost one year after randomization (48). Efficacy patterns from single-agent anti-CTLA4 therapy, including concerns about the confounding effects of pseudoprogression, led to recommendations to delay initial radiographic assessment and/or consider relatively minor tumor growth not to represent treatment failure, as captured in immune-related response RECIST guidelines (49). However, it has become clear that PD1/PDL1-directed therapies, which account for the overwhelming majority of ICI administration in the present study and in clinical practice more broadly, exhibit response dynamics similar to other types of cancer treatment (50). Specifically, tumor bulk does not appear to hinder efficacy, pathologic responses may occur within one month of treatment initiation, radiographic response is reliably associated with survival, and survival curves separate relatively early in the course of treatment (51, 52). Additionally, although use of corticosteroids to treat irAE is generally not associated with worse clinical outcomes (53), the relative timing of irAE and corticosteroid use has not been thoroughly evaluated and remains an area of interest for future studies.

Strengths of our study include detailed clinical data abstraction, ample clinical follow-up, and controlling for the opportunity to develop future irAE in landmark analyses. Although patients included in the analysis were treated off-protocol with standard of care ICI, imaging study performance and timing were relatively consistent and comparable to patterns seen in clinical trials. Although irAE rates in the present study exceeded those generally reported in clinical trials, they resembled those reported in real-world populations (54). The timing of irAE onset was also comparable to other reports, including registrational ICI trials (27). Limitations include the single-center setting, multiple cancer and ICI types, limited sample size that precluded subgroup analyses (such as type of cancer, ICI, or irAE), and the absence of tumor- and patient-related predictive variables such as PD-L1 expression, tumor mutational burden, steroid use, antibiotic exposure, and line of therapy. Nor do we have data on ICI withholding, rechallenge, or discontinuation. Lastly, we recognize that our findings may not be applicable to clinical situations in which treatment efficacy is not assessed frequently, such as prolonged adjuvant therapy for melanoma, kidney cancer, and lung cancer.

In conclusion, unlike patterns seen with other types of systemic cancer therapies, the temporal relationship between ICI efficacy and toxicity is complex and bidirectional. Neither parameter can be routinely relied on to predict the future behavior of the other. Given these observations, stringent monitoring for irAE in all patients throughout and possibly even after therapy should be encouraged, and efforts to identify irAE predictive biomarkers remain critically important to the optimal use of cancer immunotherapies.

## Data availability statement

The raw data supporting the conclusions of this article will be made available by the authors, without undue reservation.

## Ethics statement

The studies involving humans were approved by UT Southwestern Institutional Review Board. The studies were conducted in accordance with the local legislation and institutional requirements. The participants provided their written informed consent to participate in this study.

## Author contributions

MV: Conceptualization, Data curation, Investigation, Writing – original draft, Writing – review & editing. YY: Data curation, Formal analysis, Investigation, Methodology, Visualization, Writing – original draft, Writing – review & editing. YW: Data curation, Formal analysis, Writing – review & editing. DH: Data curation, Writing – review & editing. TS: Data curation, Investigation, Methodology, Visualization, Writing – review & editing. FF: Project administration, Supervision, Writing – review & editing. VP: Data curation, Writing – review & editing. MA: Data curation, Writing – review & editing. JH: Resources, Writing – review & editing. JD: Resources, Writing – review & editing. SR: Resources, Writing – review & editing. JL: Resources, Writing – review & editing. HH: Resources, Writing – review & editing. RH: Resources, Writing – review & editing. TW: Formal analysis, Investigation, Methodology, Supervision, Writing – review & editing. YX: Writing – review & editing, Methodology, Project administration, Supervision. DG: Conceptualization, Funding acquisition, Investigation, Project administration, Resources, Supervision, Visualization, Writing – original draft, Writing – review & editing.
