# Supplementary material for: Highly variable timing renders immunotherapy efficacy and toxicity impractical biomarkers of one another in clinical practice
Source: Front Immunol. 2024 Apr 16;15:1351739. doi: 10.3389/fimmu.2024.1351739 (PMC11058939; doi:10.3389/fimmu.2024.1351739)

**Supplemental Figure 1**. Timing of baseline imaging study in relation to ICI initiation.


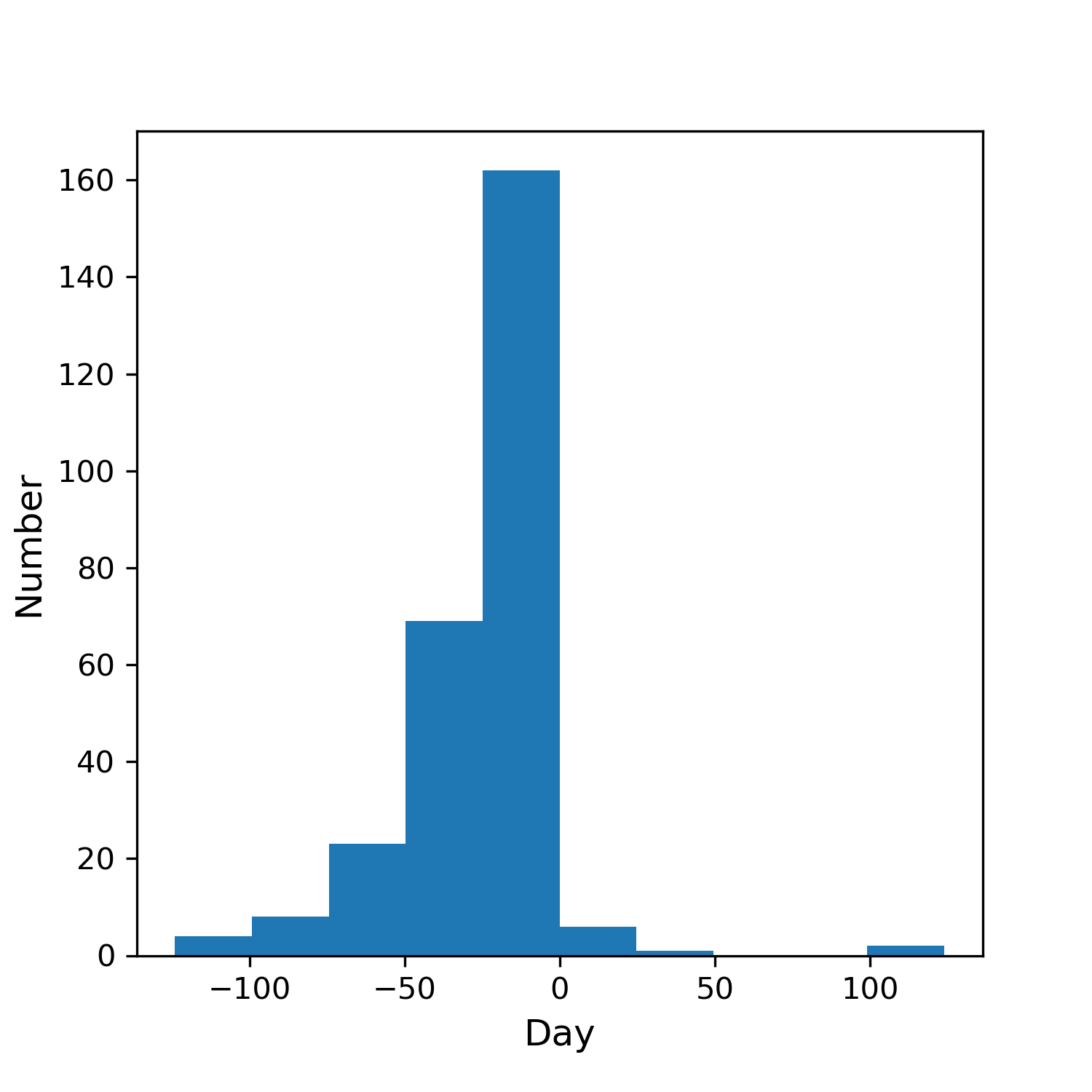


**Supplemental Figure 2**. Interval between baseline imaging study and initial radiographic assessment.


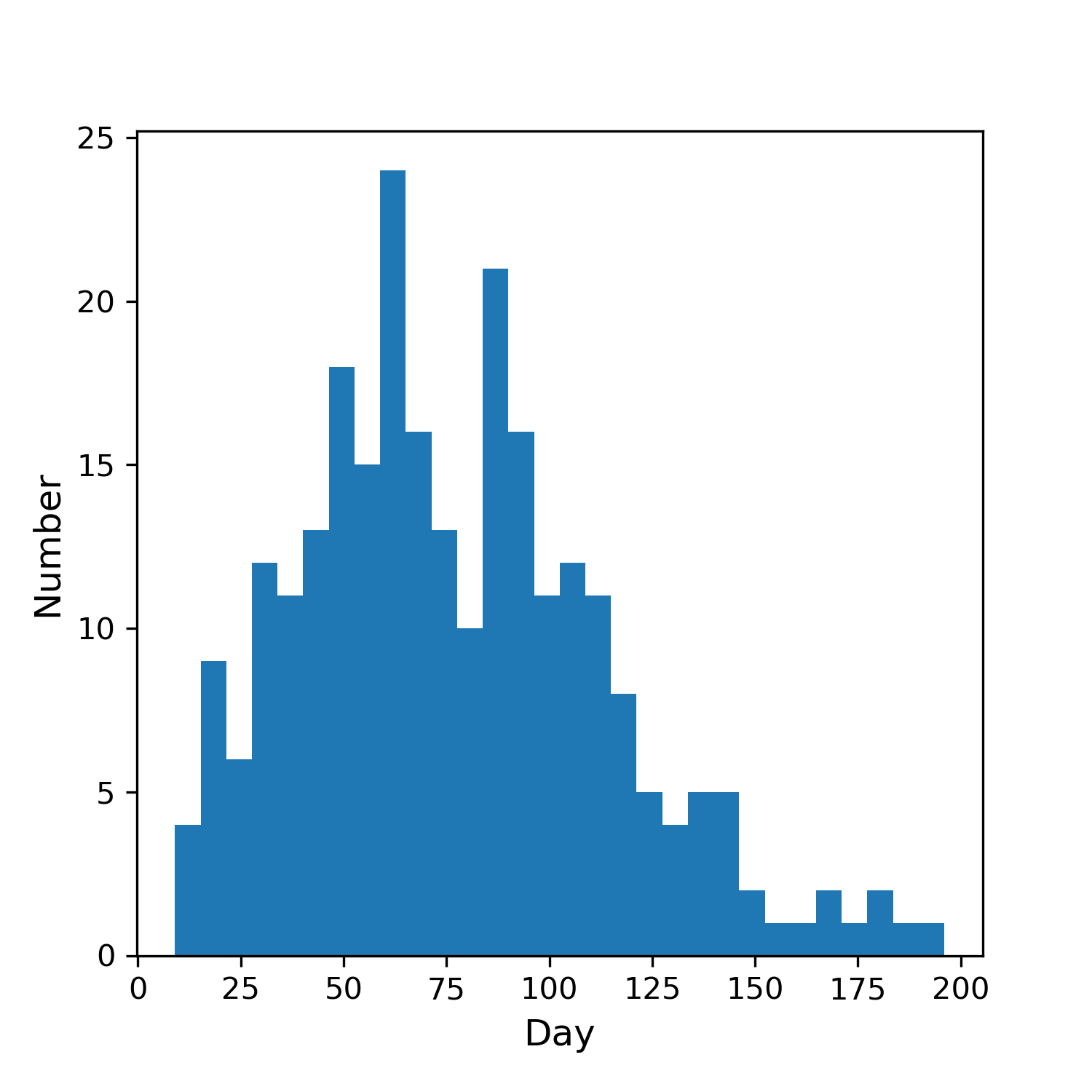

Supplement: Supplementary file 1 [file DataSheet_1.docx]
